# Substitution and Imitation

**Published:** 1902-06

**Authors:** 


### Substitution and Imitation.

THE importance of obtaining from the dispenser the precise article, drug, or preparation, prescribed by the physician has never been questioned. It is the underlying factor of all successful therapeutics. It is the safeguard of both physician and patient.

In these latter days in which there is such intimate relationship between physician, druggist and manufacturing pharmacist, the interests of these three are closely allied in the application of therapeutics to diseased conditions. Whenever, therefore, the prescriber fails to secure purity of drug, or fixedness of standard in the manufactured preparation, the reputations of all these three are liable to suffer.

This subject has been discussed in general or in particular time and again, principally through the columns of medical journals. We have been led to refer to it this time because of three or four recent violations of the ethical principles herein presented. Within the last month a New York druggist has been detected in refilling the bottles which had contained a certain water, well known in the market, with ordinary Croton water, thus practising deceit and fraud of which he was duly convicted.

Some time ago the Farbenfabriken of Elberfeld Company detected a band of drug counterfeiters in the most despicable practice of adulterating the chemicals, which are placed upon the market through the agency of this well-known house. The products, themselves, were not only imitated but the labels and containers were also counterfeited. The firm prosecuted the parties and they were duly convicted; but it behooves druggists and physicians to be on their guard when buying or prescribing aristol, phenacetin, sulfonal, trional and other similar chemical products lest they, perchance, come across the adulterated articles. The firm has protected itself by its prompt action against further criminal practice of this kind, and the court of last resort has affirmed the judgment of the court below.

A still more recent flagrant and contemptible fraud has been unearthed by the M. J. Breitenbach Company of New York, the distributing agents of Gude's pepto-mangan. It appears that a Boston firm copied the style and wrapper of the package used in putting up the well-known preparation of iron and manganese above referred to. The firm was brought into court in Massachusetts and upon conviction a decree was issued from which the following excepts are taken:

It is ordered, adjudged and decreed, that the defendant, ————, its directors, officers, agents and servants, be and they hereby are enjoined from making or using in any way, the terra cotta colored wrapper with white letters thereon, and the package in connection therewith, heretofore used by the defendant for its preparation of iron and manganese, a specimen of which wrapper is annexed to the Bill of Complaint and marked "B," or any other wrapper or package therewith which imitates the wrapper used by the complainant, the M. J. Breitenbach Company for its Gude's pepto-mangan, a specimen of which wrapper is annexed to the Bill of Complaint marked "A,' and from selling or offering for sale any preparation of iron and manganese in any package or wrapper of a terra cotta color with white letters of the same style, shape, and general arrangement as those of the aforesaid wrapper used by the plaintiff, the M. J. Breitenbach Company, and from using the words "peptonate-manganese" on or in connection with such terra cotta colored wrappers with white letters of the same style, shape, and general arrangement, as those of exhibit "A."

And the defendant is directed to forthwith deliver to the plaintiff or its attorneys, to be destroyed, all the terra cotta colored wrappers and packages aforesaid like the said exhibit "B," which the defendant now has on hand or in stock or under its control in any way.

And it is further ordered, adjudged and decreed, that the defendant, ————————, account to the plaintiff, the M. J. Breitenbach Company, for all profits which the said defendant has made from sales of said preparation in its said wrapper and package, and for all profits which the plaintiff would have made in the sales of its Gude's pepto-mangan but for the use made by the defendant of its said wrapper and package, and also for the damages to the reputation and standing of the plaintiff's said preparation, known as Gude's pepto-mangan, by reason of the said use by the defendant of its said packages and wrappers, and of its preparation therein contained, and for the damages otherwise sustained by the plaintiff by reason of the matters charged in the complaint.

We have quoted from this decree in considerable detail for the purpose of showing how complete the remedy is through the courts in such cases, provided the aggrieved persons have the courage, as in the instance above cited, to invoke the law. It would appear that the plaintiff in this action has obtained as full redress as it is possible for the law to give. The plaintiffs in these actions deserve the congratulations of the medical profession, as well as of druggists and pharmacists, for establishing their rights so conclusively. The several states should enact laws making the penalties severe for imitation or substitution.

